# Compound Dihuang Granule Changes Gut Microbiota of MPTP-Induced Parkinson’s Disease Mice via Inhibiting TLR4/NF-κB Signaling

**DOI:** 10.1007/s11064-023-04004-9

**Published:** 2023-08-10

**Authors:** Zhu-qing He, Peng-fei Huan, Li Wang, Jian-cheng He

**Affiliations:** 1 School of Traditional Chinese Medicine, Shanghai University of Traditional Chinese Medicine, Shanghai, 201203 China; 2 Shanghai Municipal Hospital of Traditional Chinese Medicine, Shanghai University of Traditional Chinese Medicine, Shanghai, 200071 China; 3 Shanghai Key Laboratory of Health Identification and Assessment, School of Basic Medicine, Shanghai University of Traditional Chinese Medicine, Shanghai, 201203 China

**Keywords:** Parkinson’s disease, Compound Dihuang Granule, Gut microbiota, Neuroinflammation, TLR4/NF-κB pathway, Network pharmacology

## Abstract

Intestinal microbiota was connected to Parkinson’s Disease (PD) pathology. The ancient Chinese medication for PD is Compound Dihuang Granule (CDG), and we found a neuroprotective function in treating the constipation of PD patients. Nevertheless, the mechanism of action still needs to be clarified. We predicted the probable targets of CDG against PD through Traditional Chinese medicine (TCM) network pharmacology and verified the analysis through animal experiments in vivo. The protein–protein interaction (PPI) network analysis screened PD-related genes, including Toll-like receptor 4(TLR4), TANK-binding kinase 1(TBK1), Nuclear Factor- Kappa B (NF-κB), and Tumor necrosis factor (TNF). Gene Ontology (GO) and Kyoto Encyclopedia of Genes and Genomes (KEGG) analyses proved that the NF-κB and toll-like receptor signaling pathways serve a key function in CDG therapy of PD. Molecular docking analysis demonstrated that CDG strongly connected to TLR4/NF-κB. Experiments findings indicated that CDG improved the damage of dopaminergic neurons and gut microbial dysbiosis, ameliorated motor impairments, and suppressed the PD-associated inflammation and oxidative stress in mice induced by 1-methyl-4-phenyl-1,2,3,6-tetrahy dropyridine (MPTP). CDG suppressed the inflammatory proteins in the colon and protected the intestinal barrier. Overall, CDG improved gut microbial in PD by blocking the pathway of TLR4/NF-κB.

**Supplementary Information:**

The online version contains supplementary material available at 10.1007/s11064-023-04004-9.

## Introduction

Parkinson’s disease (PD) is connected to extremely high morbidity and mortality [1]. PD pathogenesis is the outcome of interacting genetic and environmental variables, and its typical pathological features are dopaminergic neurons selective degradation in the substantia nigra (SN) and Lewy body creation [2, 3]. Non-motor symptoms (NMS) of PD patients are PD prodromal manifestations, such as gastrointestinal dysfunction (dysphagia, gastric emptying delay, and constipation) that predate motor signs (static tremor, stiffness, and bradykinesia) [4, 5]. Besides being among the most prevalent non-motor signs in PD patients, constipation is a recognized feature of prodrome PD, which has a hugely adverse consequence on the survival and living standard of PD patients, hypothesizing that PD arises in the intestine [4, 6, 7].

Recently, many researchers demonstrated that intestinal microbiota is associated with PD pathology. With modern science and technologies, including 16S rDNA sequencing, and metabolomics, intestinal microbiota and brain-gut could transmit information in two directions, the “microbe-gut-brain axis” [8, 9]. Moreover, intestinal bacteria affect neural development and participate in brain function and body metabolism, and entheogenic encephalopathy serves a crucial role in neurodegenerative diseases [10]. TLR4 did a vital part in recognizing microbial components, activating NF-κB signaling, and releasing the inflammatory cytokine Interleukin-1β (IL-1β), tumor necrosis factor-a (TNF-a), Interleukin-6 (IL-6) [11, 12].

Moreover, constipation in PD patients relates to intestinal bacterial dysbiosis and α-synuclein aggregates in the enteric nervous system [13, 14]. Studies have shown gut microbiota variations between healthy controls and PD patients [15]. Bacteroides abundance decreased, Blautia and Prevotella increased, motor symptoms and constipation symptoms improved significantly in PD patients after fecal microbiota transplantation (FMT) [16]. Although the dysbiotic gut microbiota has a function in PD disease pathogenesis, the concrete mechanism is obscure. Compound Dihuang Granule (CDG) is a traditional Chinese medicine used for treating PD. It exerted a significant neuroprotective effect through multiple targets, increased dopaminergic neurons numbers and improved motor symptoms, inhibited nigrostriatal pathway apoptosis and effectively improved gastrointestinal dysfunction in 6-Hydroxydopamine (6-OHDA)-induced PD rats [17, 18]. Our previous study found that CDG decreased inflammatory factor expression NF-κB and inflammatory cytokines IL-6 and TNF-a in 6-OHDA-induced PD rat [19]. However, CDG particular mechanism of action remains obscure.

Network pharmacology is a frontier discipline that uses various omics and network analysis technologies to build and reveal the connection among drugs, components, targets, and diseases, elaborate the mechanism of drug action, and explore the correlation between drugs and diseases. This method is often used to analyze the action mechanism of multi-target medicines in complex conditions [20]. We used Cytoscape software to build a “herb-component-target-pathway” network and made molecular docking with the core active components of CDG and the core action targets. CDG might treat PD by TLR4/NF-κB signal pathway. Therefore, we focused on TLR4/NF-κB signal pathway and sought to analyze the neuroprotective role of CDG in regulating intestinal microbiota imbalance by treating mice with MPTP-induced PD and exploring its practical mechanism.

## Methods

### CDG Preparation

CDG is effective for treating PD and is composed of seven traditional Chinese medicines: Shú dì huáng (Rehmannia glutinosa (Gaertn.) DC.), Bái sháo (Paeonia lactic flora Pall), Gōu téng (Uncis Uncaria rhynchophylla (Miq.) Miq. ex Havil), Zhēn zhū mǔ (Hyriopsis cumingii (Lea)), Dān shēn (Salvia miltiorrhiza Bge), Shí chāng pú (Acorus tatarinowii Schott), Quán xiē (Buthus martensii Karsch). Herein, the English name of herbal medicines referred to the Chinese pharmacopeia, and the CDG standard utilized followed the Chinese pharmacopeia (2010 version). The CDG was prepared and subjected to quality control analysis using LC–MS spectrometry [17]. Based on the human-mouse dose-conversion formula [D_B_ (mouse) = D_A_ (human)*1/388], 9.1-fold the normal dose for an adult human was determined as the appropriate dose for the mouse models,which were orally administrated 10 g/kg/day CDG for treatment.

### Network Pharmacology Analysis

#### Screening and Predicting the Target of Active Components of CDG

The active ingredients of CDG were obtained from (http://bionet.ncpsb.org/batman-tcm/ and http:// tcmspw.com/databases). Active ingredients of CDG were selected satisfying that a drug-likeness (DL) ≥ 0.18 or score cutoff ≥ 20 in addition to an oral bioavailability (OB) ≥ 30% [17]. PD-related targets were taken from the Therapeutic Target Database (TTD; http://db.idrblab.net/ttd/), Online Mendelian Inheritance in Man (OMIM, https://omim.org/), Genecards (https://www.genecards.org/), PharmGBK (https://www.pharmgkb. org/), and DrugBank (https://go.drugbank.com/), Comparative Toxicogenomics Database (CTD; http://ctdbase.org/), DisGeNET (https://www.disgenet.org/).

#### Target PPI Network Construction

We matched the obtained CDG active components with PD-related targets, and the intersection target was considered the target of CDG treatment of PD, which was presented with a Venn diagram. Then, the shared goals of CDG and PD were loaded into STRING (https://string-db.org/), and Cytoscape (3.8.0) was used to build a protein–protein interaction (PPI) network. Meanwhile, the median values greater than EC (Eigenventor centrality), DC (Degree sentrality), CC (Closeness centrality), BC (Betweenness centrality), LAC (Local average connectivity-based method), and NC (Network centrality) were utilized as screening factors to identify the possible core target of CDG in treating PD.

#### Gene Ontology (GO) and Kyoto Encyclopedia of Genes and Genomes (KEGG) Analyses

To investigate the biological processes and mechanisms implicated in PD treatment by CDG, GO and KEGG enrichment analyses were done for candidate targets. Therefore, the finding showed that GO terms or KEGG pathways having a P-value < 0.05 were regarded to be significant.

#### Molecular Docking

Retrieve and download SDF structure (2D structure) files of main active ingredients through Pub Chem website. Download PDB format file corresponding to target protein in PDB database. The essential active ingredients of CDG were selected for molecular docking with the core target. PyMOL (2.4.0) Software was utilized to eliminate water molecules and ligands from core target proteins. AutoDock Vina software was employed to dock the receptor protein with the small ligand molecule and finally obtain its conformation.

### Experimental Validation

#### Animals and Experimental Design

Male C57BL/6 mice that were 7 ~ 8 weeks old and weighed 20 ± 2 g were acquired from Shanghai Slake Experimental Animal Co. Ltd. (Shanghai, China). Mice were fed and drank water ad libitum and kept in standard environments (at 22 ± 2 °C, humidity 55 ± 10%, 12 h light/dark cycle) in compliance with the permission of the Experimental Animal Ethics Committee of Shanghai University of Chinese Medicine (PZSHUTCM200717037).

**Fig. 1:**
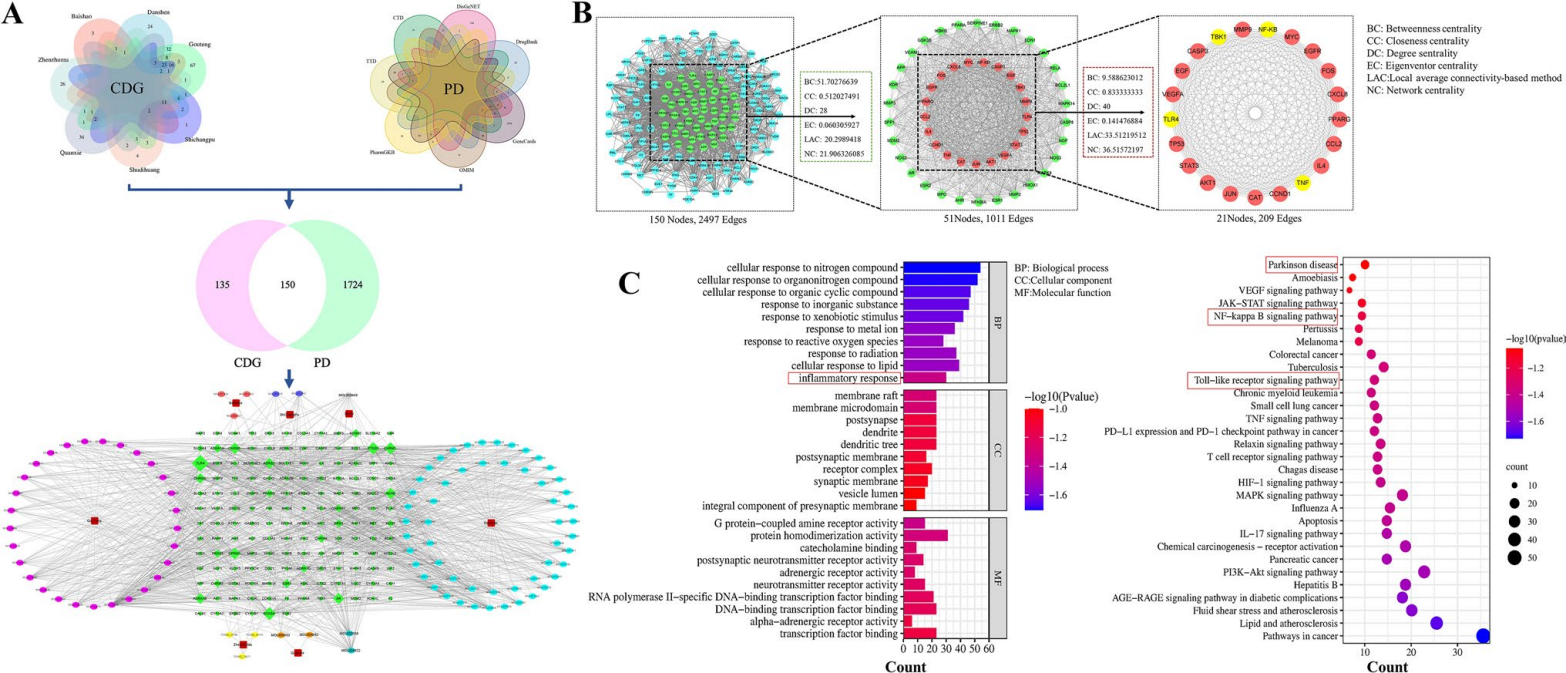
Network pharmacology and molecular docking analyses of CDG treatment in PD. **A** Developing a CDG ingredient-target network, the red squares indicate herbs; the green diamond nodes represent targets; the colored circular nodes indicate ingredients from different herbs; the big size circular is closer related to the centrality; **B** Composition-target network of CDG; 21 important targets were finally screened out; **C** Enrichment results for GO functions and KEGG pathways for CDG versus PD; the top-30 terms in every GO category and significant changes of top-30 pathways were identified according to the *P*-value < 0.05

In total, 24 C57BL/6 mice were randomly split into the control group (Normal), the model group (MPTP), as well as the treatment group (CDG; each n = 8). The model and treatment groups received 1-methyl-4-phenyl-1,2,3,6-tetrahy dropyridine (MPTP, 30 mg/kg) by intraperitoneal (I.P.) injection to construct the PD model, and the normal mice were given equal normal saline. Each group received an injection once a day for five successive days. After molding, 10 g/kg CDG was orally administrated to mice in the treated group, while the other two groups were given normal saline for seven days. Behavioral training was given for three days before behavioral testing, and after the treatment, all groups of mice underwent behavioral testing, and fresh feces and tissues were collected. Figure 1 represents the experimental flow chart.

#### Behavioral Experiments

An open field test was conducted in 30 cm × 30 cm the observation box. Prior to the experiment, the mice habituated to their surroundings for 1 h. The mice were then set in the center of the open field reaction box, and the camera recorded the movement distances of mice for 5 min [21]. After the experiment, real-time video recording analyzed the distance from the center point and the residence time.

The pole test was performed referring to a study [22]. A wooden pole with a diameter of 1.5 cm and 50 cm long, with a cork ball with a diameter of 2.5 cm fixed at the top. The mouse was placed on the top of the pole ball facing head up. The Pole test was done by training mice to reach the bottom of the pole. The turn head time and climbing time of each group were recorded. All mice were tested three times and averaged as the final result.

#### Collecting Samples and Preparing Tissue

The mice from each group were individually placed in an empty, clean high-pressure cage, and the feces were instantly gathered in sterile EP tubes and kept at − 80 °C. Tissues from the brain, colon, and serum were recovered for the study.

#### Western Blot Analysis

RIPA buffer (Beyotime, Shanghai, China) was used with 1% phenylmethanesulfonyl flfluoride (PMSF) (Beyotime, Shanghai, China) to isolate the total protein. Therefore, the homogenized protein was centrifuged at 13,000 rpm, 4 °C for 5 min to recover the total protein, and mixed with SDS loading buffer to boil for 10 min for denaturation. Total protein levels were evaluated with a BCA kit (Takara, Shanghai, China). A 30 µg of total protein was isolated using 10% sodium dodecyl sulfate–polyacrylamide gel electrophoresis (SDS-PAGE), transmitted to polyvinylidene difluoride (PVDF) membranes, then incubated at 4 °C overnight of the subsequent antibodies: Tyrosine hydroxylase:TH (1:1000; 58844, Cell Signaling Technology), TBK1 (1:1000; abs132000, Absin), NF-κB p65 (1:1000; 8242S, Cell Signaling Technology), TNF-α (1:1000; 11948, Cell Signaling Technology), TLR4 (1:1000; abs132000, Absin), Beta Actin (1:2000; 20536–1-AP, Proteintech). Therefore, incubated with secondary antibodies: anti-rabbit IgG (1:10,000; 5151S, Cell Signaling Technology) or Anti-mouse IgG (1:10000; 5470S, Cell Signaling Technology) for 1 h. The blots were discovered utilizing the LI-COR Odyssey system (Odysscy CLx, USA), and ImageJ analyzed the densities.

#### Immunohistochemistry (IHC)

Brain tissues were dehydrated at 20% sucrose. After tissue sinking, the 30% sucrose solution was dehydrated and sectioned. Brain tissue was cut into 30 μm thickness and selected from SN was blocked in 10% BSA 37 °C for 1 h after that incubated with TH (1:1000, sc-25269 AC, Sigma). Then, ABC universal mini-plus kit (ZG0615, VECTOR) was utilized. Positive cells number were measured using ImageJ.

#### Immunofluorescence (IF) Staining

The tissue of the brain and colon at 30 μm thickness was frozen. Then, sections were incubated with 0.3% BSA in 5% Triton X-100 at room temperature (RT) for 1 h. Sections were incubated at 4 °C overnight with primary antibodies: TH (1:500; sc-25269 AC, Sigma), TNF-α (1:1000; 11948, Cell Signaling Technology), TLR4 (1:1000; abs132000, Absin), Glial fibrillary acidic protein: GFAP (1:1000; 80788S, Cell Signaling Technology), Ionized calcium binding adapter molecule 1: Iba-1 (1:400; 019-19741, Wako), Zonula occluden 1:ZO-1 (ab96587; Abcam), then incubated with secondary antibodies anti-rabbit IgG (1:1000; 4412, Cell Signaling Technology) or Anti-mouse IgG (1:1000,4409S, Cell Signaling Technology) for 1 h at 37 °C. A confocal microscope was used to observe the images, and Image J was used to calculate positive cells.

#### Enzyme-Linked Immunosorbent Assay (ELISA)

Shanghai Weiao Biotechnology Company offered the ELISA kit to examine the expression of superoxide dismutase (SOD), malondialdehyde (MDA), interleukin-1β (IL-1β), and TNF-α in the mice serum. The experimental methods were conducted.

#### 16S rDNA Microbiota Profiling

DNA from mice fecal samples was extracted using the CTAB. 16S rDNA sequencing was done on the NovaSeq PE250 platform (Illumina, San Diego, CA, USA). The V3-V4 regions on bacterial 16S rDNA genes were amplified with forward (F341F5′-CCTACGGGNGGCWGCAG-3′) and reverse (805R 5′-GACTACHVGGGTATCTAATCC-3′) primers [23]. We conducted first denaturation at 98 °C for 30 s; 32 cycles of denaturation at 98 °C for 10 s, annealing at 54 °C for 30 s, and extension at 72 °C for 45 s; and a final extension at 72 °C for 10 min as PCR conditions amplify prokaryotic 16S fragments. The PCR products were purifyied by AMPure XT beads (Beckman Coulter Genomics, Danvers, MA, USA) and quantified by Qubit (Invitrogen, USA). The amplicon pools were prepared for sequencing and the size and quantity of the amplicon library were assessed on Agilent 2100 Bioanalyzer (Agilent, USA) and with the Library Quantification Kit for Illumina (Kapa Biosciences, Woburn, MA, USA), respectively. Experiments, including DNA extraction and detection, PCR amplification, product purification, library construction, and high-throughput sequencing, were done utilizing Lc-Bio technologies (Hangzhou, China).

#### Bioinformatics Analysis

Three parameters (Chao1, Shannon, and Simpson) were used in the QIIME program. Alpha diversity was applied in analyzing complexity of species diversity for a sample through Chao1 index and was calculated with QIIME2. QIIME2 estimated beta diversity. PCoA plots of beta diversity based on weighted UniFrac analysis in different groups [24]. Besides, the analysis of similarity (ANOSIM) method was performed to compare the group differences.We used the SILVA (Release 132; https://www.arb-silva.de/documentation/release-132/) and the NT-16S database for the classification of species in addition to subsequent evaluation to confirm the accomplishment and accuracy of the annotation results. Utilizing R (v3.5.2), a principle coordinates analysis (PCoA), heatmap analysis, Bray–Curtis similarity clustering, as well as species abundance analysis, were conducted, and were analyzed by the Kruskal–Wallis.

#### Statistical Analysis

GraphPad Prism8 software reported experimental data as mean ± standard deviation (SD). Findings were measured utilizing one-way analysis of variance (ANOVA) or non-parametric Kruskal–Wallis test, with a *P* < 0.05 showing a significant difference.

## Results

### Network Pharmacology Research

#### Screening Targets and Active Ingredients of CDG

Herein, active ingredients from CDG were finally screened from the related database, and 285 targets were obtained after removing and merging (Fig. 1A, File S1). Moreover, 1874 disease targets were obtained from the databases of Drangbank, PharmGKB, GeneCard, CTD, OMIM, DisGeNET, and TTD (Fig. 1A, File S2). The Venn diagram shows the obtained 150 CDG and PD intersection targets (Fig. 1A, File S3).

#### PPI Network Analysis of CDG Treatment for PD

To explore the interaction between 150 common targets, we imported them into the STRING data analysis network to build a PPI network and visualized on Cytoscape (Fig. 1A). We finally obtained 21 core targets that played an important role in PD, including TLR4, TBK1, NF-κB, and TNF (Fig. 1B, File S4).

#### GO and KEGG Enrichment Analyses

Based on *p*-value screening, we selected the top 30 items of GO and KEGG analyses based on the *P*-value (Fig. 1C, File S5). According to the screening results, BP mainly involved the response to reactive oxygen species and cellular response to lipid, inflammation response. CC analysis found that it mainly included an integral component of the presynaptic membrane, vesicel lumen. MF analysis demonstrated that the targets were primarily included in the transcription factor binding and alpha-adrenergic receptor activity. CDG might exert therapeutic PD by participating in multiple biological processes. Through KEGG enrichment analysis, the pathways involving core targets were obtained, including the NF-κB and Toll-like receptor signal pathways (Fig. 1C, File S6). Moreover, the findings also demonstrated that the target genes of CDG active components were strongly connected to PD and linked to other diseases, including tumors, lipids, atherosclerosis, and infectious diseases, suggesting the potential advantages of CDG in treating these diseases.

#### Molecular Docking

**Fig. 2:**
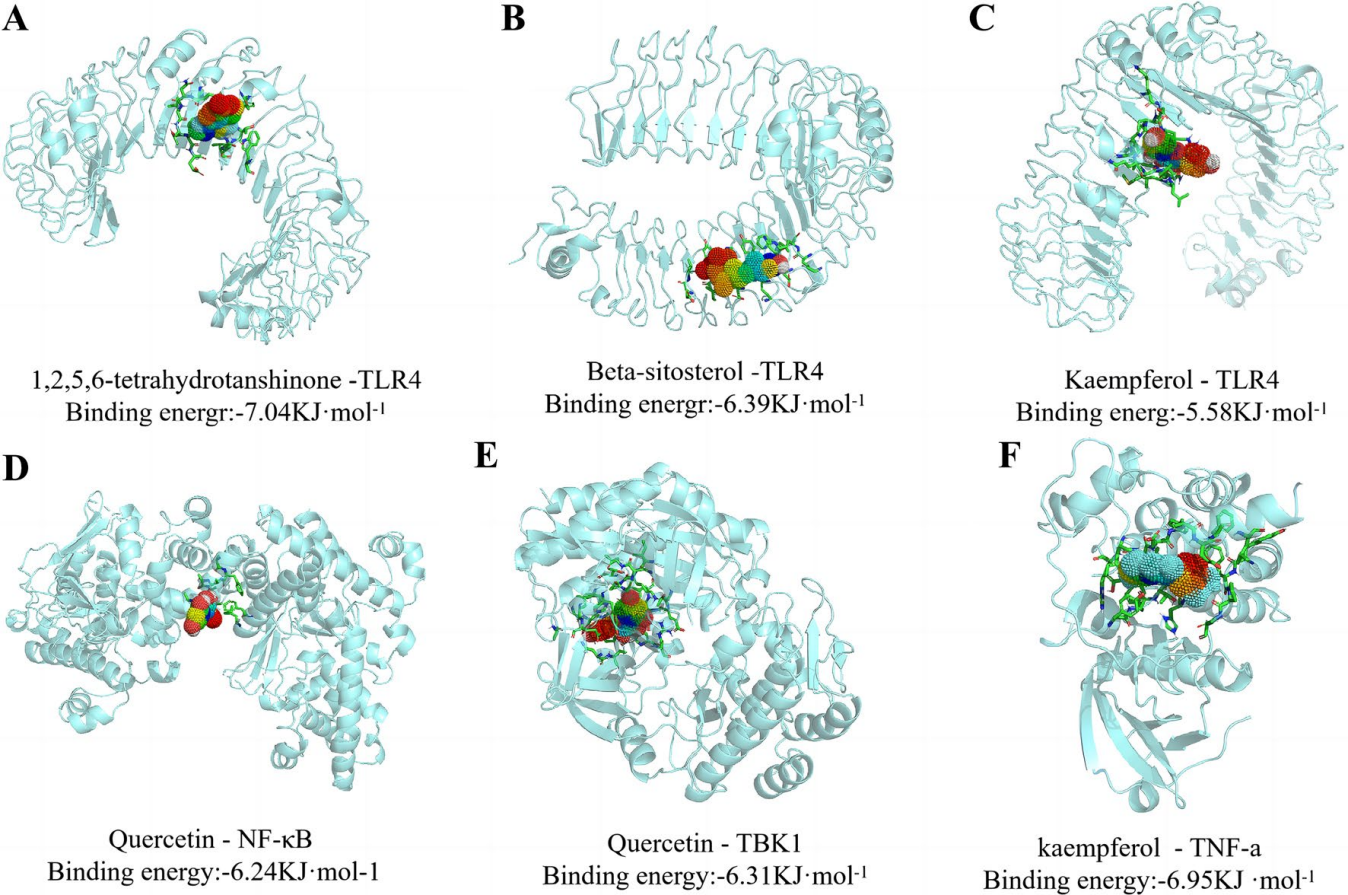
Docking of active ingredients with TLR4/NF-κB. **A** 1,2,5,6-tetrahydrotanshinone -TLR4; **B** Beta-sitosterol-TLR4; **C** Kaempferol-TLR4; **D** Quercetin-NF-κB; **E** Quercetin-TBK1; **F** kaempferol—TNF-a

Figure 2 illustrates findings of molecular docking; the screened active components 1,2,5,6-tetrahydroptanshinone, Beta-sitosterol, and Kaempferol had good binding energy with TLR4 (Binding energy: − 7.04 kJ·mol^−1^, − 6.39 kJ·mol^−1^, − 5.58 kJ·mol^−1^; Fig. 2A–C). Quercetin had a good combining ability with NF-κB and TBK1 (Binding energy: − 6.24 kJ·mol^−1^, − 6.31 kJ ·mol^−1^, Fig. 2D–E). Moreover, kaempferol had a good binding ability to TNF-a (Binding energy: − 6.95 kJ mol^−1^, Fig. 2F).

### Experimental Validation

#### CDG Improved Motor Functions in MPTP-Induced PD Mice by Avoiding Dopaminergic Neuronal Death

**Fig.3:**
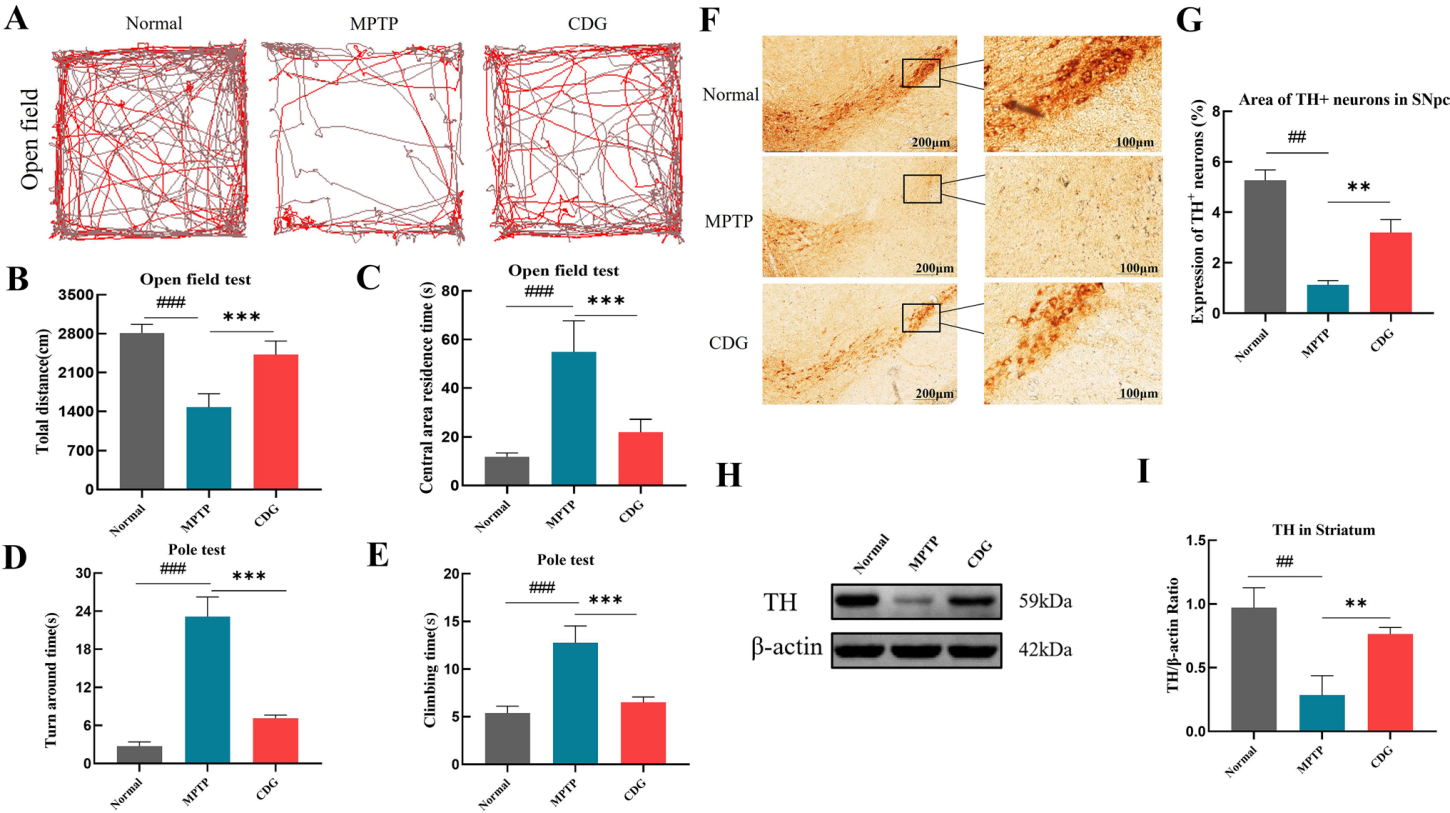
CDG treatment increased dopaminergic neuronal and improved motor functions in MPTP-induced PD mice. **A** Mice autonomous activity trajectories were recorded during the Open field exam; **B** Total motor distance of the mice in five minutes; **C** Stay time in the central area; **D** Mice turn around time during the Pole test; **E** Mice climbing time during the Pole test; **F** IHC staining of Tyrosine hydroxylase(TH) in SN; the scale bar is 200 μm; 100 μm; **G** TH^+^ neuron cells count in SN; **H** TH expression in the Striatum with WB; **I** The outcome of TH WB density analysis in Striatum; one-way analysis of variance was employed to evaluate the data; n = 8 for the behavioral test; n = 3 for WB and IHC; findings are shown as mean ± SD, vs. Normal; ^###^*P* < 0.001; vs. MPTP; ***P < 0.001

Motor disorders and dopaminergic neurons injury are commonly present in PD models. Open field and pole tests were performed on animals to evaluate the possible neuroprotective effect of CDG on the motor function of MPTP-induced mice. Open field test demonstrated that motor dysfunction in the MPTP group was reflected in the reduced total motor distance and increased dwell duration in the central area (*P* < 0.001, *P* < 0.001, MPTP vs. Normal; Fig. 3A–C). CDG significantly improved the exercise ability and exploration ability of the PD mice (*P* < 0.001, *P* < 0.001, CDG vs. MPTP; Fig. 3B–C). Pole test results found that PD mice exhibited a significantly reduced capacity for grip ability, showing a significantly longer head-turning time and pole climbing time (*P* < 0.001, *P* < 0.001, vs. Normal), and CDG treatment improved motor function of MPTP-induced PD mice (*P* < 0.001, *P* < 0.001, vs. MPTP; Fig. 3D–E).

Moreover, we further examined alterations of dopaminergic neurons in SN, and TH expression, which is a dopaminergic biomarker. Immunohistochemical staining findings noted that the shape of TH-positive cells became blurred, and TH-positive cells expression was also decreased significantly when contrasted with the normal group mice (*P* < 0.001), and the TH expression was significantly raised after CDG treatment (*P* < 0.01, Fig. 3F–G). Meanwhile, Western blot (WB) analysis with anti-TH antibody validated the above results (*P* < 0.01, vs. Normal; *P* < 0.01, vs. MPTP, Fig. 3H–I).

#### CDG Improves the Gut Microbial Dysbiosis of MPTP-Induced PD Mice

**Fig.4:**
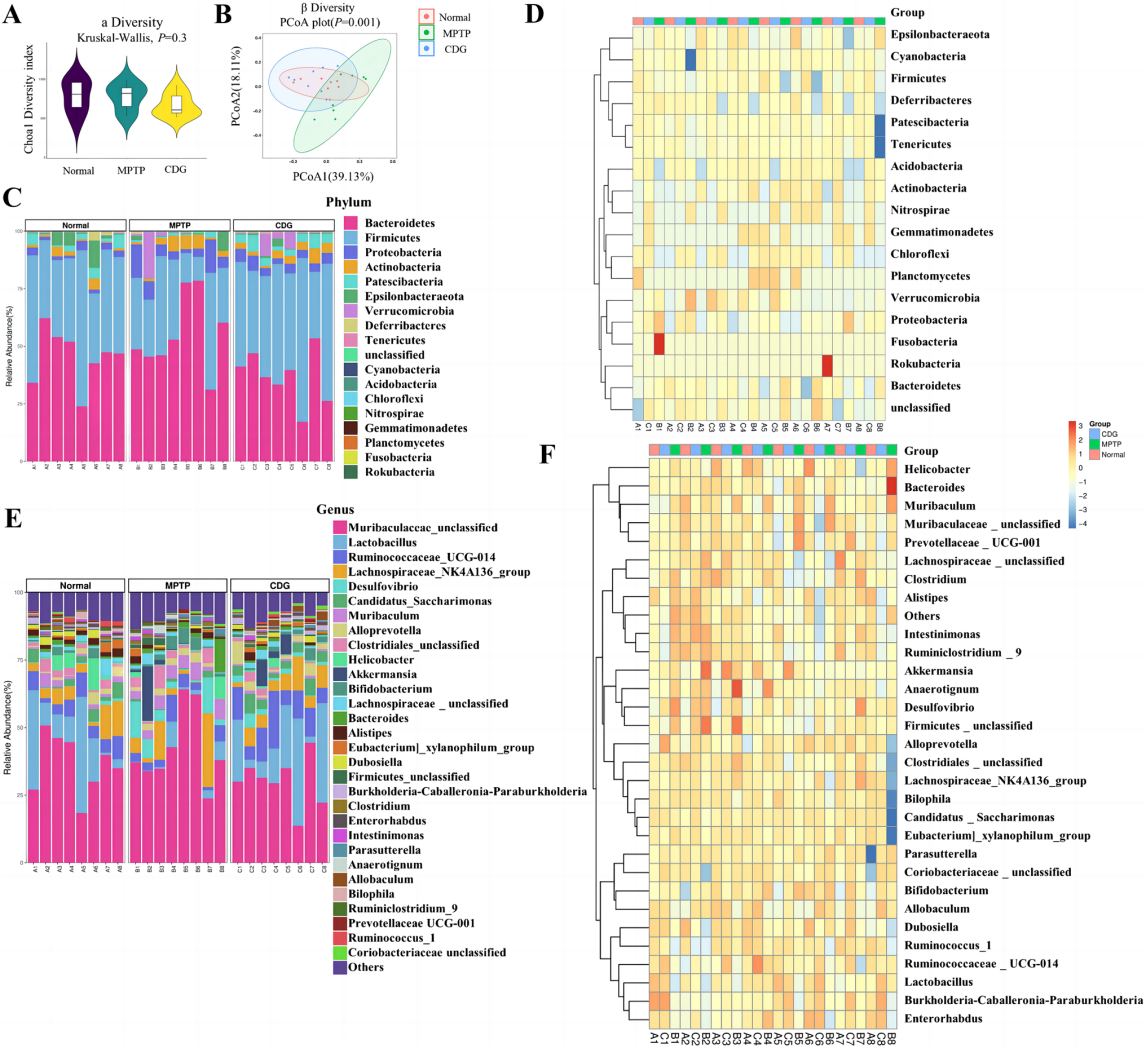
CDG treatment improved the dysbiosis of gut microbes of MPTP-induced PD mice. **A** Chao1 analysis of the alpha diversity of gut microbiota; **B** Beta diversity analysis in different groups; Principal coordinates analysis (PCoA)1 and 2 explain 39.13% and 18.11% of the variance, respectively. Different groups are highlighted with different colors. The position and distance of data points indicates the degree of similarity in terms of both the presence and relative abundance of bacterial taxonomies. **C** Relative predominance of gut microbiota at the Phylum level in each group; **D** Heatmap analysis of relative predominance of gut microbiota at the Phylum level for various groups; **E** Relative predominance of gut microbiota at the Genus level for each group; **F** Heatmap analysis of relative predominance of gut microbiota at the Genus level in various groups; the data were analyzed with Kruskal–Wallis; n = 8

We observed the species abundance, evenness, and intestinal microbiota distribution among the mice, including alpha and beta diversity analysis by 16S rDNA sequencing. Figure 4A depicts non-significant differences between the three groups by the alpha diversity indices (Chao1, File S7). The results showed the microbial composition difference between the three groups (Fig. 4B, File S8). We analyzed the species composition and differences in the samples to further identify the potential bacterial groups of microbial dysbiosis. We presented the abundance and composition of each group using a heatmap (Fig. 4D and F, File S9). At the phylum level, the dominating bacteria were Bacteroidetes, Firmicutes, and Proteobacteria (Fig. 4C). We found a significant increase in the predominance of Proteobacteria, Patescibacteria in the MPTP group (*P* < 0.01, *P* < 0.01 vs. Normal), and reduced after CDG administration (*P* < 0.01, vs. MPTP; Table. 1). At the genus level, the dominating bacteria mainly included Muribaculaceae_unclassified, Lactobacillus, and Ruminococcaceae_UCG-014 (Fig. 4E). PD mice showed decreased Lactobacillus, Bilophila, Candidatus_Saccharimonas, and Ruminococcaceae_UCG-005 at the genus level (*P* < 0.05, *P* < 0.01, *P* < 0.05, *P* < 0.05, vs. Normal), and increased Lactobacillus, Ruminococcaceae_UCG-014, Candidatus_Saccharimonas, Enterorhabdus after CDG therapy (*P* < 0.05, *P* < 0.01, *P* < 0.01, *P* < 0.05, vs. MPTP; Table. 1). Meanwhile, the predominance of Muribaculum, Turicibacter, Desulfovibrio were raised at genus in PD mice (*P* < 0.01, *P* < 0.05, *P* < 0.01, vs. Normal. CDG mice showed a lowered predominance of Muribaculum and Turicibacter (*P* < 0.001, *P* < 0.05, vs. MPTP; Table. 1).

**Table 1:** Microbiota relative predominance at various taxon levels

Taxonomic level	Relative predominance			P value		
	Normal	MPTP	CDG	N.vs.M	M.vs.C	N.vs.C
Phylum						
Firmicutes	42.92 ± 12.85	28.93 ± 13.97	47.40 ± 13.41	0.269	0.044*	1.000
Proteobacteria	2.41 ± 1.04	6.974 ± 4.97	4.30 ± 1.35	0.006**	1.000	0.059
Patescibacteria	0.08 ± 0.05	0.02 ± 0.02	0.06 ± 0.03	0.001**	0.034*	0.977
Genus						
Lactobacillus	15.81 ± 15.58	2.835 ± 3.45	18.33 ± 14.72	0.040*	0.019*	1.000
Ruminococcaceae_UCG-014	5.73 ± 2.47	4.21 ± 2.74	10.4 ± 5.39	0.730	0.009**	0.214
Muribaculum	1.92 ± 1.44	4.55 ± 1.95	0.81 ± 0.27	0.002**	0.000***	0.128
Candidatus_Saccharimonas	2.864 ± 2.11	0.72 ± 0.81	4.06 ± 1.79	0.046*	0.002**	0.337
Desulfovibrio	0.52 ± 0.50	5.39 ± 5.33	1.98 ± 1.45	0.003**	1.000	0.054
Bilophila	0.72 ± 0.73	0.12 ± 0.22	0.43 ± 0.31	0.007**	0.065	1.000
Turicibacter	0.08 ± 0.08	0.17 ± 0.28	0.85 ± 0.71	0.692	0.011*	0.006*
Enterorhabdus	0.54 ± 0.31	0.31 ± 0.32	0.69 ± 0.24	0.262	0.041*	0.580
Ruminococcaceae_UCG-005	0.12 ± 0.11	0.03 ± 0.06	0.03 ± 0.02	0.040*	1.000	0.257

Statistical comparative using one-way ANOVA with Holm-Sidak post-hoc comparisons

The findings reflect the means = SD; 77 = 8, *P < 0.05, **P < 0.01, ***P < 0.001

#### CDG Ameliorated Neuroinflammation and Oxidative Stress in MPTP-Induced PD Mice

**Fig.5:**
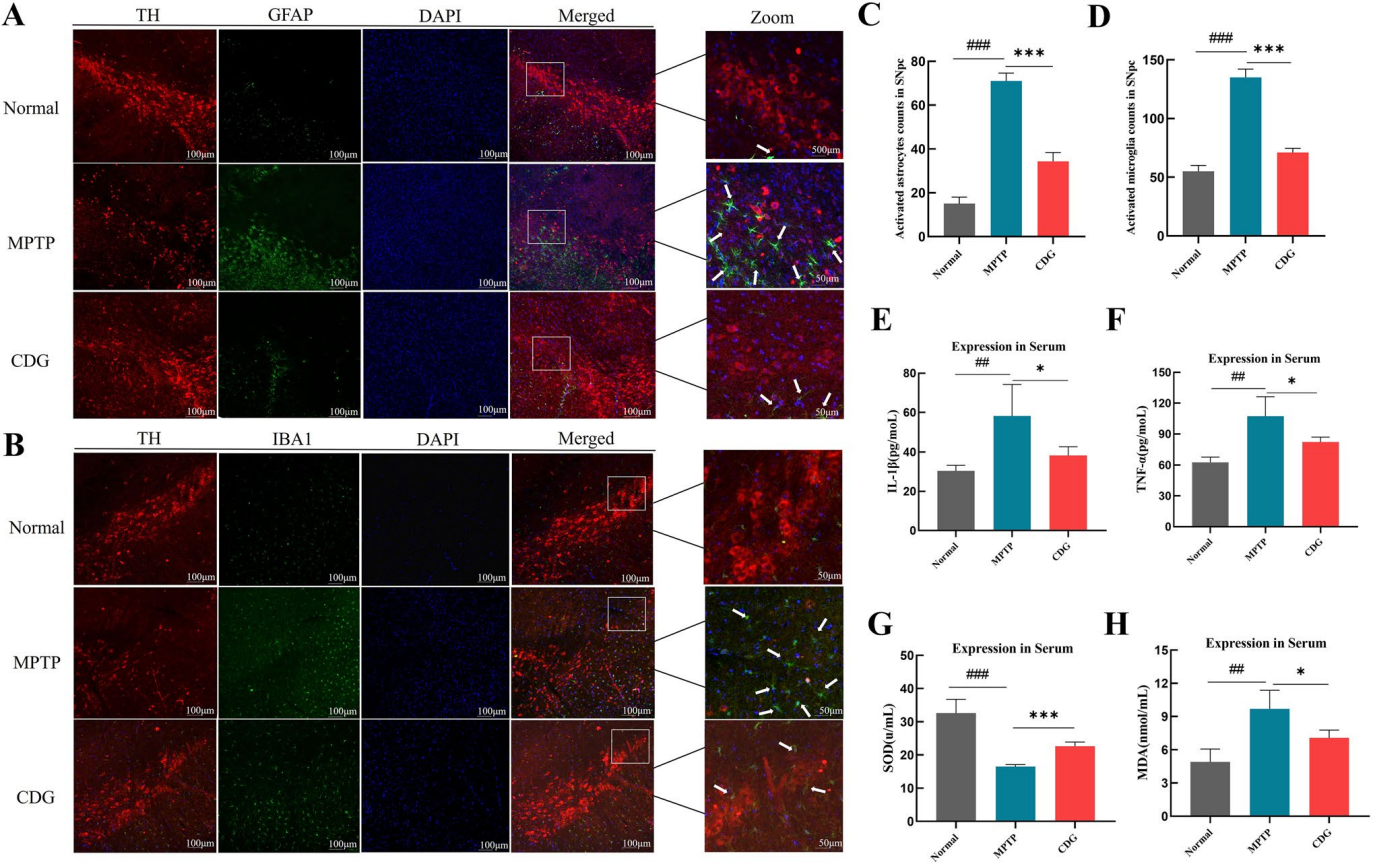
In MPTP-induced PD mice, CDG treatment suppressed the PD-associated inflammation and oxidative stress. **A** IF staining for TH (Red) and GFAP (Green) in the SNpc. **B** IF staining for TH(Red) and IBA1 (Green) in the SNpc; **C**, **D** the proportion of activated astrocytes and microglia in the SNpc; **E**, **F** IL-1 β; TNF-α serum expressions; **G**, **H** SOD; MDA serum expressions; white arrows represent the positive cells in SNpc; scale bar is 100 μm; 50 μm; findings are assessed with a One-way ANOVA; n = 3 for IF; n = 4 for ELISA; findings are presented as mean ± SD, VS Normal; ^##^*P* < 0.01; ^###^*P* < 0.001; vs. MPTP; *P < 0.05; ***P < 0.001

Given that gut dysbiosis could promote inflammation as well as oxidative stress in the brain of PD patients and trigger the excessive response of the immune system accompanied by systemic inflammation [11]. Glial activation is strongly associated with PD neuroinflammation development [25, 26]. We observed the expression of GFAP and Iba1 (green) with TH (Red) colocalization in SN neurons utilizing immunohistochemistry staining. The findings demonstrated that GFAP and Iba1 expressions showed significant upregulation after MPTP injury in model group mice (*P* < 0.001, *P* < 0.001, vs. Normal, Fig. 5A–D), and we found a significant decrease in GFAP and Iba1 expressions after CDG therapy than the model group (*P* < 0.001, *P* < 0.001, vs. Normal, Fig. 5A–D). Concurrently, the morphological changes in GFAP^+^ and Iba1^+^ cells were found. The data above demonstrated that CDG reduced both GFAP and Iba1 increase and ameliorated the neuroinflammation in MPTP-induced PD mice.

Utilizing ELISA, the experimental findings demonstrated that inflammatory factor IL-1β and TNF-α expressions were lowered significantly after CDG treatment (*P* < 0.05, *P* < 0.05, vs. MPTP; Fig. 5E–F). We also assayed two related genes expression associated with oxidative stress, SOD and MDA. We observed a significant rise in MDA level and decrease in SOD activity in PD mice (*P* < 0.01, *P* < 0.001, vs*.* Normal), CDG treatment lowered MDA level and raised SOD activity (*P* < 0.05, *P* < 0.001, vs. MPTP; Fig. 5G–H). The above experimental findings proved that CDG ameliorated the neuroinflammation and oxidative stress in MPTP-induced PD mice.

#### CDG Suppresses Colonic Inflammation and Intestinal Leakage in MPTP-Induced PD Mice

**Fig. 6:**
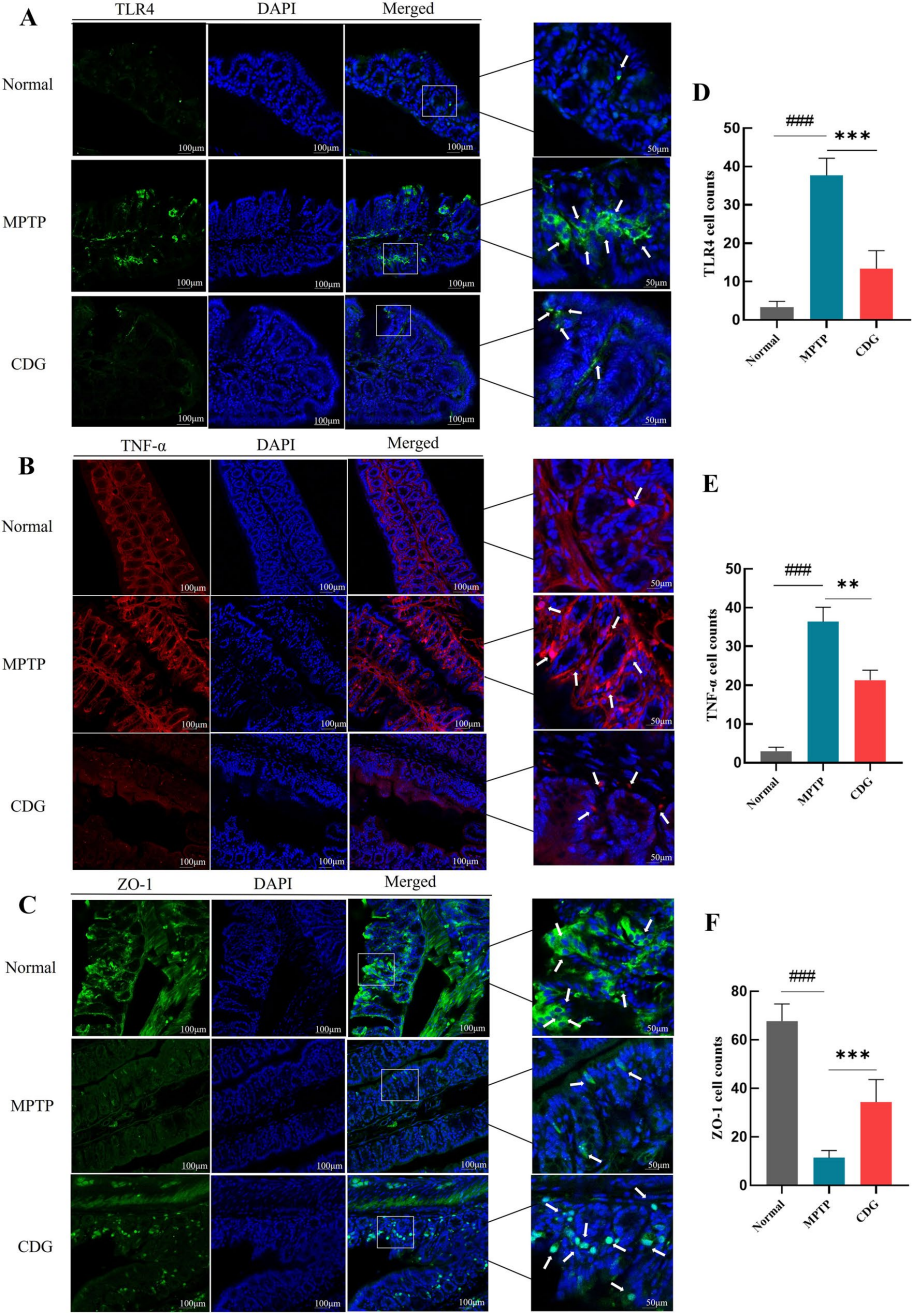
CDG treatment suppressed the inflammatory proteins in the colon and protected the intestinal barrier in MPTP-induced PD mice. **A**–**C** IF staining for TLR4(Green); TNF-α (Red); and ZO-1(Green) in the colon; **D**–**F** the numbers of TLR4; TNF-α; and ZO-1 cells in the colon; white arrows present the positive cells in the colon; the scale bar was 100 μm; 50 μm; the data are assessed utilizing a One-way ANOVA; n = 3 for IF; findings are demonstrated as mean ± SD; vs. Normal, ^###^*P* < 0.001; vs. MPTP, **P < 0.01, ***P < 0.001

Usually, gut dysbiosis may result in intestinal permeability and activate TLR signaling. We further examined whether CDG modulated gut integrity and examined the expression of the key mediator TLR4 and inflammatory factor TNF-α in colonic tissue. IF assay showed that TLR4 and TNF-α cells expression increased in PD mice colon (P < 0.001, P < 0.001, vs. Normal) and significantly decreased after CDG therapy (P < 0.001, P < 0.01, vs. MPTP; Fig. 6A, B and D, E). Gut barrier integrity is directly associated with tight junction protein expression (ZO-1) in intestinal tissue [24]. ZO-1 cells were continuously expressed in the colon of normal mice, and the intestinal mucosal integrity of PD mice was destroyed, and ZO-1 expression significantly lowered (P < 0.001, vs. Normal), its expression raised significantly after CDG therapy (P < 0.001, vs. MP TP; Fig. 6C and F).

#### CDG Regulated Gut Flora and Ameliorated Neuroinflammation via Inhibiting TLR4/NF-κB Pathway in MPTP-Induced PD Mice

**Fig.7:**
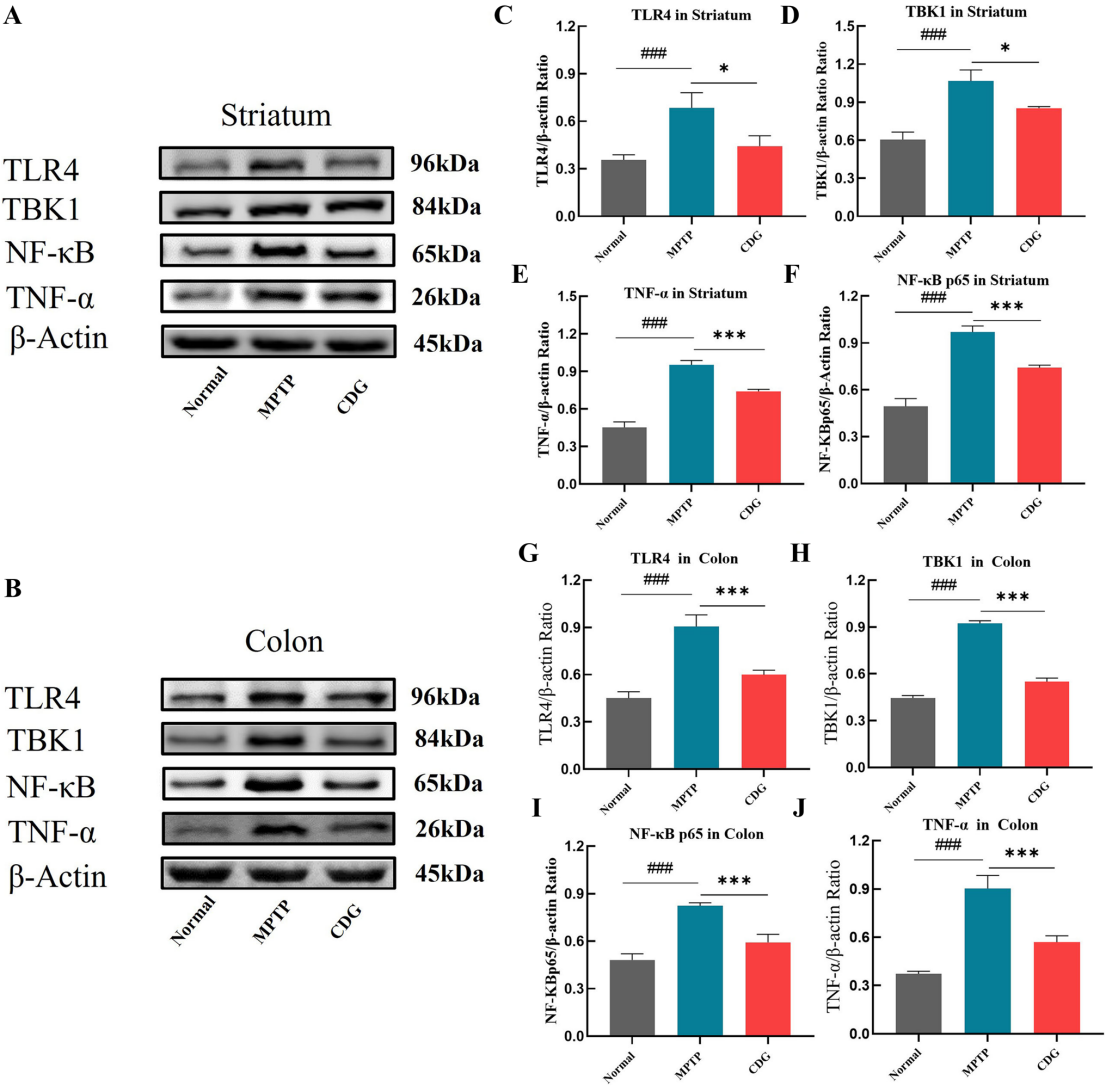
CDG treatment alleviated intestinal inflammation and neuroinflammation via blocking TLR4/NF-κB pathway in MPTP-induced PD mice. **A** Representative WB brands of TLR4; TBK1; NF-ΚB; and TNF-α in the striatum; **C**–**F** the density analysis results of TLR4; TBK1; NF-κB; TNF-α in the striatum; **B** representative WB brands of TLR4; TBK1; NF-κB; and TNF-α in the colon; **G**–**J** the density analysis results of TLR4; TBK1; NF-κB; TNF-α in the colon; findings are assessed with a one-way ANOVA, n = 3 for WB; findings are reported as mean ± SD, VS Normal; ^###^*P* < 0.001; vs. MPTP; *P < 0.05; ***P < 0.001

To determine if TLR4/NF-κB signaling was a potential target of CDG in regulating gut microbiota and ameliorating neuroinflammation, we evaluated the TLR4/NF-κB signaling expression-associated proteins in colonic and striatum tissue using WB. Findings revealed that TLR4 expression, TBK1, NF- κB p65, and TNF-α proteins increased significantly in the striatum of PD mice (*P* < 0.001, *P* < 0.001, *P* < 0.001, *P* < 0.001, vs. Normal), and the above indices were all significantly decreased with CDG therapy (*P* < 0.05, *P* < 0.05, *P* < 0.001, *P* < 0.001, vs*.* MPTP; Fig. 7A and C–F). In parallel, TLR4, TBK1, NF-κB p65, and TNF-α protein expressions were significantly raised in PD mice colon (*P* < 0.001, *P* < 0.001, *P* < 0.001, *P* < 0.001, vs. Normal); and TLR4, TBK1, NF- κB p65, and TNF-α expression exhibited a significant reduction in the colon after CDG treatment (*P* < 0.001, *P* < 0.001, *P* < 0.001, *P* < 0.001, vs. MPTP; Fig. 7B and G–J) The above results indicated that CDG might regulate gut microbiota and reduce the inflammation level by reducing the pathway of TLR4/NF-κB.

## Discussion

Currently, there are no truly ideal methods for optimal targeted drug treatments for PD. It has become a research hotspot for decreasing toxicity and side effects of anti-PD medications and finding alternative drugs. TCM showed its advantages in preventing and treating PD. CDG was developed associated with clinical experience in treating PD. To further study the CDG mechanism in treating PD, we screened the key active components of CDG in treating PD utilizing network pharmacology and found that the CDG mechanism in treating PD could be closely associated with the Toll-like receptor and NF-κB pathways. TLR4 play a crucial role in innate immunity and a dysregulation in their signaling was implicated in PD [11]. NF-κB is a one of the most important downstream molecules in the TLR4 signaling pathway [27]. Some findings suggest that gut microbiota may trigger immune activation of the colonic mucosa through the TLR4 signaling pathway, leading to neuroinflammation and subsequent neurodegeneration in PD [28]. Therefore, we focus on the TLR4/NF-κB Pathway for investigation. The following animal experiments will verify essential proteins expression on TLR4/NF-κB signal pathway and further explore the potential molecular pathway of CDG treatment of PD.

Our previous study demonstrated that CDG played a significant neuroprotective role in the MPTP-induced PD model and the 6-OHDA-induced PD model in vivo and could effectively improve gastrointestinal dysfunction and suppress microglia activation in PD mice [17, 18]. Gut microbiosis is a potential factor in PD pathogenesis, and that gut microbiota was connected to PD progression by the gut-brain axis interaction [29, 30]. Herein, intraperitoneal MPTP injection was used to establish a PD mouse model related to intestinal microbiota disorders to measure CDG neuroprotective effect on PD and further study its treatment mechanism.

PD research has thoroughly studied MPTP, among the highest-known neurotoxins disrupting dopaminergic neurons [31]. Due to its lipophilicity, MPTP might pass the blood–brain barrier, transforming it to MPDP^+^ by monoamine oxidase B in astrocytes. MPP^+^ is the active compound of dopamine neurons that enters the SNpc. It suppresses complex I of the mitochondrial electron transport chain, resulting in reduced ATP, oxidative stress, as well as degeneration of dopaminergic neurons [32–34]. Herein, MPTP-induced PD mice exhibited motor dysfunctions in open-field and pole tests. Oxidative stress was crucial in PD development and might induce degeneration of dopaminergic neurons. SOD, a radical scavenger, and MDA, a lipid peroxidation metabolite, are important indicators revealing oxidative stress levels [35, 36].

Moreover, PD mice had increased MDA and decreased SOD expression in serum, which had an oxidative stress response. IHC of brain tissue and WB showed dopaminergic damage. Together, MPTP-induced mice had PD pathology and motor symptoms. Furthermore, MPTP-induced PD mice exhibited gut microbiota dysbiosis [24, 37, 38]. Therefore, we analyzed the composition of the gut microbiota in PD mice by 16 s rDNA sequencing. Moreover, beta diversity suggested a remarkable change in the microbiota composition of mice at the phylum and Genus level. Proteobacteria and Patescibacteria abundance increased significantly in PD mice at the phylum level. Proteobacteria were associated with gastrointestinal inflammation, with higher content in PD patients, and increased Proteobacteria abundance in PD mice with MPTP molding [15, 24], consistent with our animal experiments. Lactobacillus is a probiotic bacterium that is reduced in PD patients and has also been reduced in MPTP-induced mice [5, 39].

Additionally, we first found that Genus Muribaculum and Candidatus_Saccharimonas were associated with the PD, with increased Muribaculum and decreased Candidatus_Saccharimonas in MPTP-induced PD mice. As per our results, the decreased Genus Bilophila abundance was associated with PD. Bilophila abundance is correlated with Hoehn and Yahr stages, revealing that Bilophila can serve as a progression indicator in PD [40]. Desulfovibrio can produce hydrogen sulfide and LPS, which may induce the aggregation of α-synuclein, with a higher abundance as observed in PD patients [41], also increased at Genus herein. Briefly, MPTP-induced mice had gut microbiota dysbiosis, and the mechanisms of intestinal microbiota in PD progression still need further exploration.

Further analysis of the microbiota revealed that reestablishing normal gut microbiota could enable the protective effect of CDG therapy. CDG increased the abundance of Phylum Firmicutes in PD mice. Firmicutes can produce butyrate, which is involved in the inflammatory response of body [42]. SCFAs have crucial roles in promoting intestinal function and anti-inflammatory effects. Ruminococcaceae_UCG-005 and Ruminococcaceae_UCG-014 can produce SCFAs and exert their anti-inflammatory effects [43, 44]. Herein, Genus Ruminococcaceae_UCG-005 was reduced in PD mice, and Genus Ruminococcaceae_UCG-014 was increased after CDG treatment, adjusting for the microbiota of PD mice. Moreover, the abundance of beneficial bacteria Enterorhabdus was increased after CDG administration. Turicibacter is associated with disordered lipid metabolism and can exert anti-inflammatory effects, and Turicibacter is positively associated with SCFA production [45]. Herein, the predominance of Turicibacter was raised in the PD mice after CDG administration. The increase of Turicibacter in the model group might be associated with the PD immunomodulation of mice. Overall, our data supported that CDG significantly altered some inflammation-associated microbiota in MPTP-induced mice, increasing beneficial bacteria.

Gut microbiota and its metabolites can participate in PD pathophysiology by regulating CNS neuroinflammation, gut inflammation, and barrier function through gut-brain axis interactions [46]. Dysbiosis of gut microbes might cause bacteria to produce harmful metabolites, such as lipopolysaccharides (LPS), which could disrupt the functionality of gastrointestinal barrier. TLR4 is the primary receptor for LPS immune recognition, widespread express on the surface of several cells, including microglial, astrocyte, and intestinal epithelial cells. Its activation triggers the host inflammatory response and the proinflammatory factors, including TNF-α, IL-1β, and IL-6 [47–49]. These findings revealed that gut microbiota might drive the colonic mucosa immunological activation via the TLR4 signaling pathway, resulting in neuroinflammation and further neurodegeneration in PD [50]. To further explore the mechanism by which CDG exerts neural protection by replenishing the intestinal microbiota of PD mice, we highlighted the relationship of inflammation between the gut and the brain. Microglia and astrocytes are crucial in maintaining neuronal function and brain homeostasis and are also the primary cells mediating innate immunity in the CNS [51, 52]. When activated, they secrete large quantities of inflammatory chemokines, including TNF-α and IL-1β [53]. Astrocytes can recognize the TLR mediating inflammatory CNS disease injury [47].

Our study found that microglia and astrocytes were activated in the SNpc of PD mice and were inhibited after CDG administration. Moreover, we labeled TLR4 and TNF-α cells with IF to assess intestinal inflammation. CDG reduced the infiltration of TLR4 and TNF-α cells in PD mice colonic tissue. We also discovered that cytokines (TNF-α, IL-1β) were raised in PD mice serum, suggesting that proinflammatory factors may penetrate the systemic circulation and cause a systemic inflammatory response. Gut microbial dysbiosis-mediated inflammation leads to the intestine in high permeability to cause “intestinal leakage.” Gut barrier integrity is strongly associated with tight junction protein ZO-1 expression in intestinal tissue [54]. ZO-1 expression is lowered in PD mice, and ZO-1 expression is reduced in inflammatory bowel disease (IBD) patients [55, 56]. We examined ZO-1 expression in the mouse colon utilizing IF assay and found that CDG protected the intestinal barrier via restoring tight-junction function. All the data showed that CDG could reconstruct the intestinal microbiota of PD mice to reduce microglia and astrocytes activation, inhibit serum inflammation, and protect the damaged intestinal barrier to play a neuroprotective role.

NF-κB activating factor TBK1 is a crucial factor of NF-κB and has a key part in the inflammatory immune reaction [49, 57]. Inflammatory cytokines stimulate NF-κB activation in TLR4 signaling through heterodimer RelA (p65) and p50, which translocate to the nucleus and interact with NF-κB target sites in immune response genes [49]. After TLR4 ligand binding, NF-κB signaling is activated to discharge proinflammatory cytokines (TNF-α and IL-1β), which has a crucial function in innate immune defense, neuronal excitotoxicity, and neurodegeneration [58]. Suppressing TLR4-mediated NF-κB signaling may be a successful approach against PD [59]. FLZ protects rotenone-induced PD mice by ameliorating intestinal dysbiosis and inhibiting TLR4/MyD88/NF- κB signaling pathway in SN and colon [60]. Herein, we further demonstrated the crucial function of activating the TLR4 pathway in the microbe-gut-brain axis involved in PD pathology. Besides reconstructing the gut microbiota dysbiosis in PD mice, CDG effectively reduced TLR4, TBK1, NF-κB, and TNF-α expressions in the PD mice striatum and colon. The molecular mechanisms of CDG in improving gut microbiota dysbiosis could be connected to inhibiting TLR4/NF-κB pathway.

## Conclusion

These experiments revealed that CDG regulated gut microbiota dysbiosis in MPTP-induced PD mice, reduced neuroinflammation, and induced neuroprotective effects via suppressing TLR4/NF-κB pathway, offering fresh perspectives for more intensive research. This study has limitations, which had a small sample size and had no clinical observation involved. In the future, we will include clinical PD patients for validation to further explore the molecular mechanisms of improving PD intestinal microbiosis and exerting neuroprotection.

### Supplementary Information

Below is the link to the electronic supplementary material.

Supplementary file1 (XLSX 11 KB)

Supplementary file2 (ZIP 493 KB)

## Data Availability

We declare that the data that support the findings of this study are available from the corresponding author upon reasonable request.
